# Motor Skill Development in Italian Pre-School Children Induced by Structured Activities in a Specific Playground

**DOI:** 10.1371/journal.pone.0160244

**Published:** 2016-07-27

**Authors:** Patrizia Tortella, Monika Haga, Håvard Loras, Hermundur Sigmundsson, Guido Fumagalli

**Affiliations:** 1 Department of Diagnostics and Public Health, Research Center on Child Motor Development, University of Verona, Verona, Italy; 2 Department of Physiotherapy, Sör-Tröndelag University College, Trondheim, Norway; 3 Department of Psychology, Norwegian University of Science and Technology, Trondheim, Norway and Reykjavik University, Reykjavik, Iceland; University of Rome, ITALY

## Abstract

This study examined the effects and specificity of structured and unstructured activities played at the playground Primo Sport 0246 in Northern Italy on motor skill competence in five years old children. The playground was specifically designed to promote gross motor skills in preschool children; in this study 71 children from local kindergartens came to the park once a week for ten consecutive weeks and were exposed to 30 minutes of free play and 30 minutes of structured activities. Before and after the ten visits, each child completed nine tests to assess levels of motor skills, three for fine-motor skills and six for gross-motor skills. As control, motor skills were also assessed on 39 children from different kindergartens who did not come to the park. The results show that the experimental group who practiced gross-motor activities in the playground for 1 hour a week for 10 weeks improved significantly in 4 out of the 6 gross motor tasks and in none of the fine motor tasks. The data indicate that limited transfer occurred between tasks referring to different domains of motor competences while suggesting cross feeding for improvement of gross-motor skills between different exercises when domains related to physical fitness and strength of specific muscle groups are involved. These results are relevant to the issue of condition(s) appropriate for maintaining and developing motor skills in this age group as well as for the planning, organization and implementation of play and physical activities in kindergartens.

## Introduction

Prevention of sedentary behavior and promotion of a physically active lifestyle are now consolidated issues of health campaigns in most of the western countries. In the Vienna declaration, the World Health Organization (WHO) has stressed the significance of physical activity (PA) for reducing preventable and avoidable mortality and disability due to noncommunicable diseases, such as cardiovascular diseases, cancer, diabetes, chronic respiratory diseases [1]. In addition, sedentary behavior has been recently associated to obesity and other noncommunicable diseases [2–4]. Accordingly, guidelines recommend that toddlers and preschoolers accumulate at least 180 min of daily physical activity at any intensity, progressing by the age of 5 toward at least 60 min of energetic play (e.g., moderate—to vigorous-intensity physical activity) [5]. In addition, the goal of minimizing the amount of time spent being sedentary for extended periods (except time of sleeping) should also be pursued [6]. Given that behavioral habits acquired in childhood typically track into adolescence and adulthood [7, 8], these recommendations are especially important for health outcomes across the lifespan [9, 10].

Driven by the health and psychological concerns related to lack of physical activity, several studies have investigated the behavior of preschool children in various environmental contexts [11–13]. Type, location and organization of spaces (indoor and outdoor) impact upon the physical activity levels of preschool children [10, 14–16]. In general, the presence of opportunities for practicing movement-based play is an important predictor of the levels of physical activity performed by the child [17]. In terms of levels of physical activity, free play appears to be less efficient than structured activities in producing high physical engagement during play [18, 19].

The level of motor skill competence may influence the amount, intensity and level of physical activity [20, 21]. For example, children with low motor competence are found to engage less in physical activities compared to their well-coordinated peers [22, 23], and to display significantly lower levels of physical fitness [24–27]. Therefore, motor competence and physical activity can be considered as interconnected concepts in child development. Finally, the ability to perform and master different types of motor skills has also been associated with school-performance [28], levels of social interaction with peers [29], and self-perception [30, 31].

In western countries, children spend several hours of the day in- kindergarten [6] and the school and their educators have a great responsibility in determining the levels of physical activity of their pupils [32]. Indeed, application of specific physical education programs can improve physical fitness and health related variables in both obese and lean preschool children [33]. Despite these evidences, a common finding is that physical activity levels are lower than recommended in preschool [10, 34, 35]. Direct observations of children in childcare showed that most of the activities were sedentary and that ≤ 3% of physical activities were of moderate or vigorous intensity [36]. In Italian kindergartens time and space dedicated to movement are very marginal and mostly related to manual fine motor skill [37]; also national guidelines for kindergarten programs do not consider physical activities and acquisition of motor skills as educational goals for educators in kindergartens [38]. In this context, a better knowledge of how specific programs and interventions can efficiently promote physical activity and development of motor skills in early childhood is required.

Accordingly, the main purpose of this study was to explore the effects and specificity of a program of physical activity performed at a specific playground in Treviso, northern Italy, on the development of motor skills in five years old children attending local kindergartens. Starting from the specific needs of the Italian preschoolers linked to the lack of attention to motor development, the playground was designed to promote development of gross motor skills related to manuality, mobility and balance. A set of objective measurement procedures assessing gross motor skills was applied before and at the end of a training period consisting in 10 visits occurring once per week to the specific playground over a period of 10 weeks. During each visit, children were exposed to 30 minutes of free play combined with 30 minutes of structured activities.

An interesting aspect of motor skills is their specificity. Accordingly, a further purpose of our study has been to investigate whether training of gross motor skills (in the domains of mobility, manuality and balance) resulted in transfer of skill acquisition between different domains and/or between gross and fine motor skills. To this aim, a set of measurements assessing fine motor skills was also included in our investigation.

## Materials and Methods

At the time of the study the University of Verona did not have an Institutional Review Board for studies that were not involving patients of the University Hospital. Accordingly, the project was examined by the Scientific Committee of Laboratorio 0246, the non-profit Association that owned the site where the research was done and has organized the activities with the schools. The Scientific Committee has approved the study and verified the adherence to the principles of the Declaration of Helsinki. Written informed consents were obtained from the parents (or guardians) before the children attended the study and from teachers; due authorizations were obtained from the directors of the schools involved. All adults received extensive written descriptions of the goals, limits and risks of the study, of the methods used and of the activities performed before being asked to sign authorizations. Field permission of the study was granted by: Laboratorio 0246—Associazione di Promozione Sociale Strada del Nascimben 1/B 31100 Treviso Phone:+39 0422 324310 Fax: +39 0422 324311 Email: info@0246.it; http://www.0246.it/. Laboratorio 0246 has made the playground available for research studies on child motor development. Its Scientific Committee, who has approved the study, has monitored the adherence to the principles of the Declaration of Helsinki throughout the study.

### Participants

The study was conducted at "La Ghirada", Treviso, Italy, a 220,000 sqm sports center based in Treviso since 1985 and owned by the Benetton Group (www.ghirada.it). La Ghirada is a multifunctional structure open for free to everybody and including the playground Primo Sport 0246, a space designed to provide controlled opportunities for practicing basic motor skills to children up to the age of six [39]. The non-profit association Laboratorio 0246 was founded in Treviso in 2011 and is based at "La Ghirada"; its aim is to support and organize research activities in the field of child development and education (www.0246.it). In collaboration with La Ghirada and the local section of the Comitato Olimpico Nazionale Italiano, Laboratorio 0246 has provided instructors to attend the children during the visits to the playground and to help collecting the data.

Four out of 23 kindergartens in Treviso, Veneto, northern Italy, were selected for participation in the study based on similarities of the socio-economical status and ethnic origin of the pupil families. Of these, two participated as experimental groups (structured and free play activities at the playground) and two as comparison groups (no activities at the playground). Of all the children enrolled in the study (104 for the experimental schools and 70 for the controls), only those that were present in all the testing sessions are considered in this study (experimental group: n = 71; 41 boys and 30 girls; mean age 5.6 ± 0.31 ys; control group: n = 39; 22 boys and 17 girls; mean age 5.7 ± 0.30 ys). Drop-out subjects were absent in one of the testing session for family or momentary health reasons.

### The Primo Sport 0246 playground

**Fig 1:**
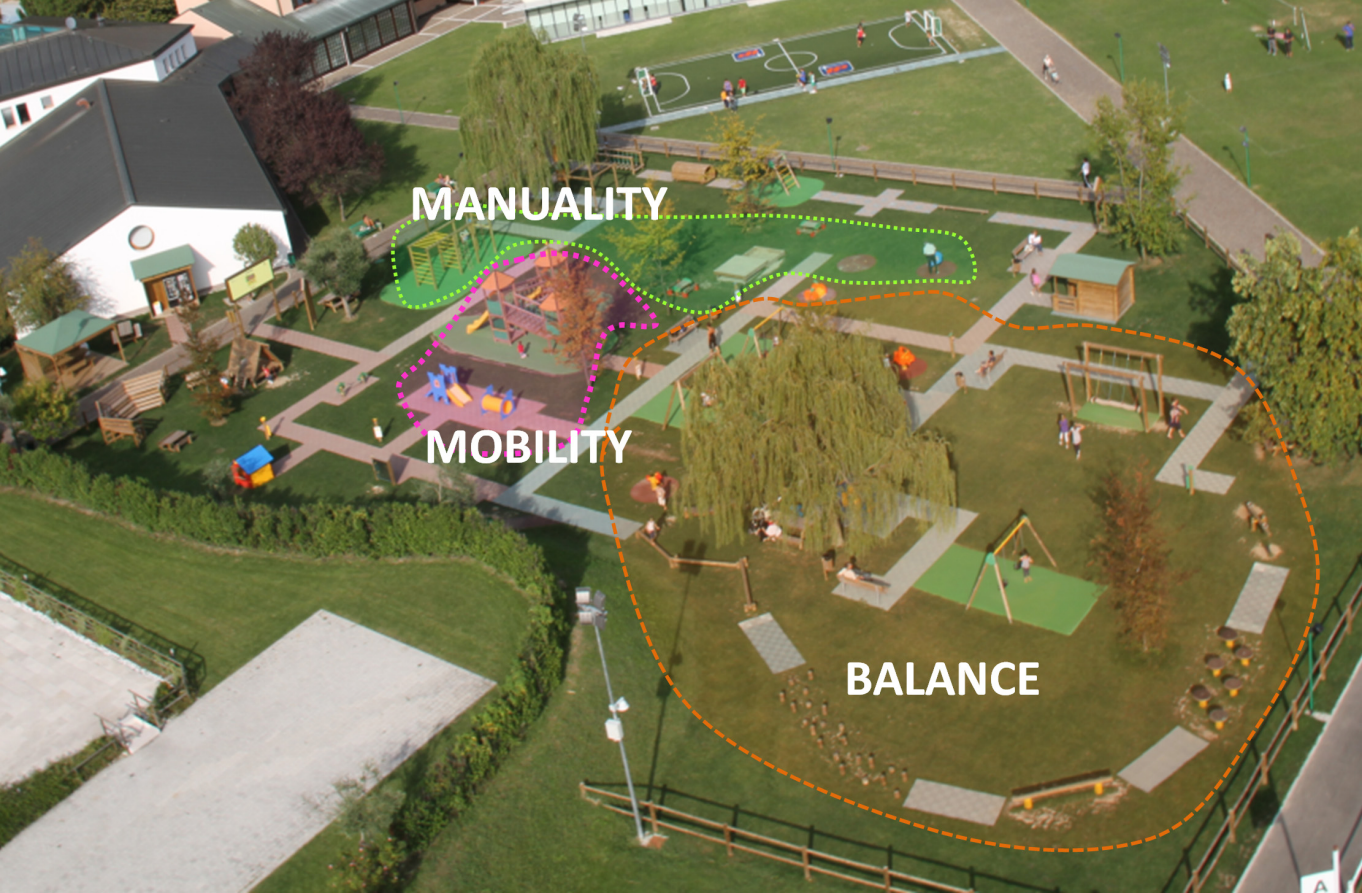
Aerial view of the playground Primo Sport 0246. The dotted lines enclose the three basic motor skill-specific areas.

The playground Primo Sport 0246 (Fig 1) is located inside "La Ghirada" and has an extension of m^2^ 2500 with a total of 35 recreational equipments placed at safety distance from each other; each equipment is endowed of a basement of consistency/softness adequate to limit traumatic damages to the user. The playground is divided in dedicated areas; in the manuality/dexterity, mobility and balance areas (limited by dashed lines in Fig 1) equipments were selected based on the motor skill most trained by their use and provided different levels of difficulties so that each child could exercise the skill regardless of his/her own level of competence (for detailed description of the organization of the park see [39]).

### Organization of activities at the playground

The playground activities consisted of 10 sessions each lasting 1 hour, executed once per week in the period Mars to May 2012. All sessions took place at either 9.00 AM or 11.00 AM, with temperatures ranging between 10 and 26 degrees Celsius; no raining days occurred during the study. Each session began with a 10–15 min walk from the bus stop to the playground. The two experimental groups went to the playground on the same day every week and the classes never met each other at the playground. During the 10 weeks, each class began session alternatively at 9.00 and at 11.00 AM (5 times each). The children of each group (maximum 40 children) were divided in two subgroups, one starting the session with the free-play time and the other with structured activities. The order of type of activity was alternated in the 10 days and each child begun his/her day at the park 5 times with free-play and 5 times with structured activities.

**Fig 2:**
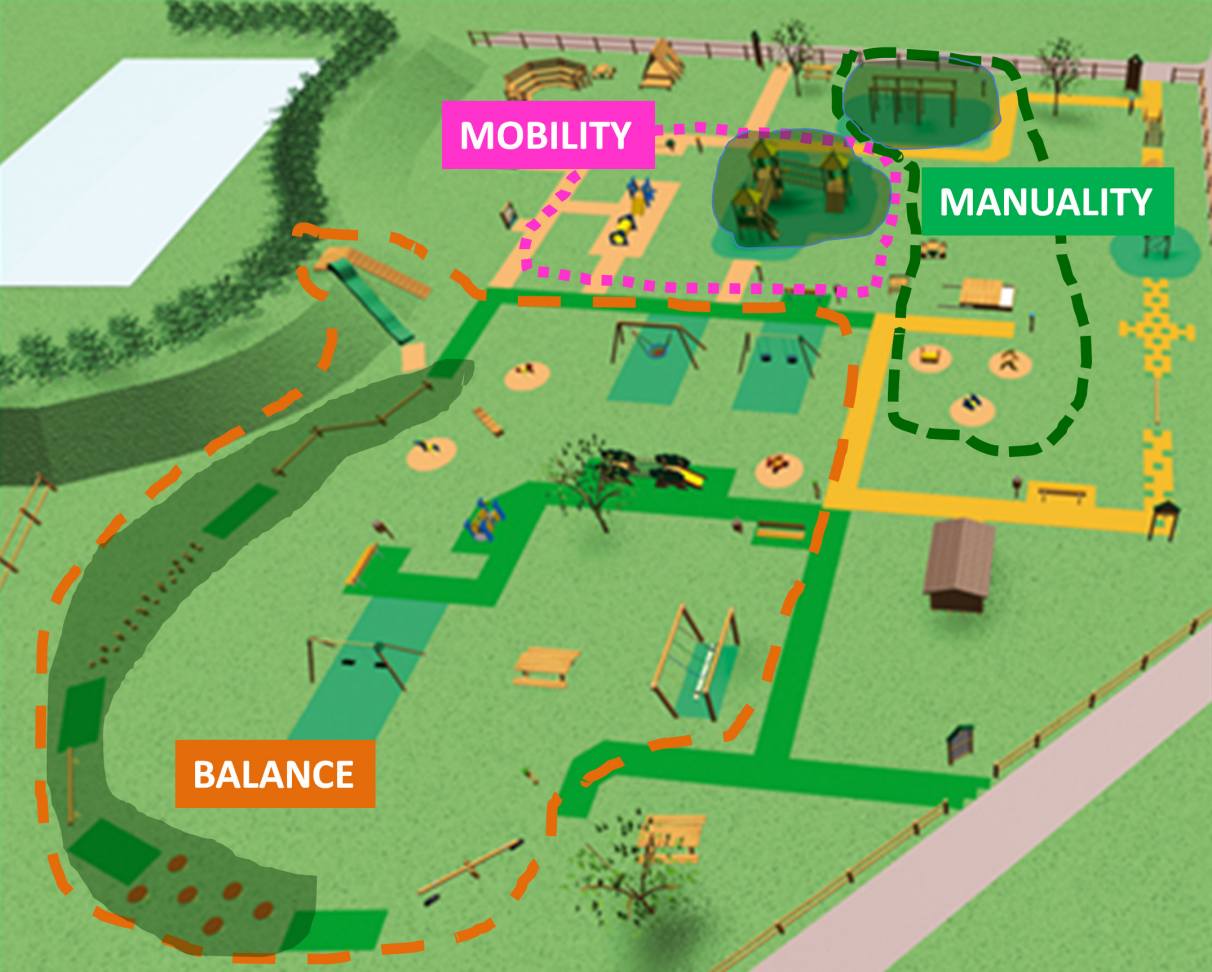
Schematic drawing of the Primo Sport 0246 playground: areas for free- and structured-activities. The darkened portions inside each of the specific areas show where structured activities were run. Free-play area was everywhere else in the park at free choice of the child. Total time for structured activities was 30 min (10 min in each of the darkened areas); time free-play was also 30 min.

During the structured activities, the children were further divided in three small groups of 6–7 children; each group spent 10 minutes in each of the three dedicated areas (manuality/manual dexterity, mobility and balance, whitened areas in Fig 2) for a total of 30 minutes. Switching from one area to the other (occurring every 10 minutes) was commanded by a field coordinator. The sequence of the activities was: 1) for manuality, use of the following tools in the sequence: rope ladder, climbing rope, hanging bar, gymnastic rings, climbing net, monkey bars; three times repetition of the circuit; 2) for balance, as above but with the following tools: balance beam, balance logs, balance elastic beam, balance platforms; 3) for mobility, each child goes up and down from various climbing points and slopes present in the mobility area and arranged as circuit; three times repetition.

Two instructors were constantly present in the manuality area, one in the mobility area and one in the balance area. The roles of the trained instructors were to provide scaffolding when asked by the child and general encouragement, to indicate the order of use of the different types of equipment and to give general instructions about their use. The instructions were given orally or by showing; for example, children were directed to walk on a balance beam and not use the equipment by crawling; on the monkey bar, the instructor showed how to move from one pole to the other. For difficult equipment, such as the elastic beam, the instructors provided encouragement, scaffolding and suggested strategies for completing the task. When a child was self-sufficient on a specific type of equipment, the instructor suggested to add new challenges, e.g. walking backward on the balance beam, running faster along slopes, increase the number of brachiations.

Another instructor controlled the time spent in each area and coordinated the switch of the groups from one area to the other.

Free-play was allowed everywhere within the playground, except for the portions of the areas where the other group was performing the structured activities (non-whitened areas in Fig 2). Time for free play was 30 min, during which schoolteachers (at least one every 10 children) were present for assistance and in case of emergency, but were not involved in the children activities.

### Assessment of motor skills

Measurements were taken at the beginning of the study and at the end after the children of the experimental group had experienced the ten visits at the playground Primo Sport 0246.

Two tasks were selected from the Test of Motor Competence: Building Bricks and Heel-to-Toe Walking [40]; four tasks were selected from the Movement Assessment Battery for Children (One leg balance right, One leg balance left, Posting Coins right and Posting Coins left) [41] and one task (Putting a Medicine Ball) was selected from the Test of Physical Fitness [42]. Two tasks, balance on beam and balance on platform, were developed as a part of the project in order to assess aspects of dynamic balance. Similar test items also appear in the Körperkoordinationstest für Kinder: KTK [43].

Assessments of motor skills were performed individually in a quiet room in the kindergarten or at the playground, and were conducted in accordance with the assessment manuals. Each test item was explained and demonstrated and each child could familiarize with the tests with a trial, before measurements were taken. Participants were given verbal encouragement and support throughout the testing procedure.

The Building Bricks and the Posting Coins (right or left hand) tests were used for fine motor skills, whereas to assess gross motor skills we used: One leg balance–right or left, Balance on beam, Balance on platforms, Heel-to-Toe Walking and Putting a Medicine Ball.

#### Building Bricks task

Twelve square-shaped Duplo™ bricks were used to build a tower as fast as possible. The participant holds one brick in one hand and one brick in the other. At a signal the participant assembles the bricks together one after one until all 12 have been put together. Neither of the arms was allowed to rest on the table and the assembled bricks were held in the air all the time. Performance was measured by the time to complete the task.

#### Posting Coins task

At the start signal the child was asked to pick up from the table 12 coins, one at a time, and to drop them through the slot in a box as quickly as possible using one hand (either the right or the left). Output is the time in seconds required to put all the coins in the box. Distinct measurements were taken for right or left hand.

#### One leg balance task

The child stands on either the right or left foot with the arms held freely at the sides. The test is performed with eyes open. The time starts when one foot leaves the floor and stops if one of the following faults occurs: moving the standing foot, the heel or the toe from its original place, touching the floor with the free foot, winding/hooking the free leg round the standing leg. Independent measurements were taken for balance on left or right leg

#### Balance on Beam task

The child begins with the feet parallel 10 cm from the beam (height at beginning: 26 cm, height at end: 13 cm, width: 13 cm, length: 300 cm; Legnolandia, Italy, cod 011065). At a start signal the participant goes up and walks as fast as possible on the beam. The time is stopped when the participants arrives at the end of the beam. Time of execution and number of errors (every time the child goes down of the beam) are recorded.

#### Balance on Platforms task

Each platform consists of a wooden disc (diameter: cm 54) supported at cm 43 of height by a metal spring that makes it very unstable (Legnolandia, Italy, Jumpy, cod. 011107). The circuit consists of 6 platforms separated by gaps of cm 60. The child begins with the feet parallel near the first platform; at the start signal the child walks on the first platform and then jumps from platform to platform to the end of the circuit. The time is stopped when the participants jumps down from the last platform. Time in seconds needed to complete the circuit was measured.

#### Heel-to-Toe Walking task

This task is often called the tandem walking test and is considered a measure of dynamic balance capabilities. The children were required to walk down a straight line (4.5 m) as fast as they could, placing their heel against the toes of the foot in each step (tandem). Performance was time to complete the line.

#### Putting a Medicine Ball

The child holds the medicine ball (diameter 20 cm, weight 1 kg; Giodicart, Italy, Cod. 5401, type Trial) against the chest while standing with feet parallel and a shoulder width apart. The ball is thrown with both hands simultaneously and the distance from the starting position to where the ball lands is measured. The better of two attempts is the test item score.

### Data Analysis

Data were analyzed by two-way ANOVA for factors TIME (pre vs post) and TREATMENT (experimental vs control) followed by post-hoc Bonferroni test. The data were analyzed using GraphPad Prism 6 software (GraphPad Software Inc., La Jolla, CA, USA); graphs were produced with Microsoft Office Excel 2007.

## Results

The subjects of the experimental (Exp) group and the controls (Con) were similar for height, weight and BMI (Exp: height 1.14 ± 0.05 m, weight 20.53 ± 3.04 Kg, BMI 15.82 ± 1.50; Con group: height 1.17 ± 0.04 m, weight 21.71 ± 4.11 Kg, BMI 15.83 ± 1.59; mean ± SD of respectively n = 71 and 39 subjects).

Data on gross-motor skills are shown in Table 1; data on fine motor skills are in Table 2.

**Table 1:** Evaluation of gross motor skills in 5 y old children.

Test		Pre-training	Post-training	Difference between post- and pre-training	*p* of Pre vs Post	*p* ofExp vs Con at post-training
***Putting a Medicine Ball (cm)***	Exp	192±44	229±50	37±66	<0.0001	<0.001
	Con	191±45	192±48	1±37	NS	
***One leg balance-Right (sec)***	Exp	13.64±9.88	18.37±14.83	4.73±15.50	<0.05	NS
	Con	13.34±9.32	16.19±13.94	2.85±14.44	NS	
***One leg balance-Left (sec)***	Exp	12.46±6.42	20.48±5.81	8.16±7.21	<0.0001	<0.04
	Con	14.47±2.80	17.15±3.39	3.16±5.59	NS	
***Balance Beam (sec)***	Exp	15.81±5.30	9.29±3.16	-6.53±5.58	<0.0001	<0.001
	Con	15.11±6.45	12.84±5.96	-2.27±4.40	NS	
***Balance on Platforms (sec)***	Exp	35.12±23.57	10.47±2.75	-24.65±22.59	<0.001	<0.0001
	Con	27.22±14.00	24.19±13.30	-3.03±12.67	<0.05	
***Heel-to-Toe Walking (sec)***	Exp	34.74±13.66	33.14±11.38	-1.60±15.31	NS	<0.001
	Con	34.04±15.23	43.70±11.52	9.66±14.79	0.001	

*Abbreviations*: Exp = experimental group; Con = control group; NS = not significant; p = Bonferroni’s test. All data are expressed as mean±standard deviation. Raw data can be found in S1 Table

**Table 2:** Evaluation of fine motor skills in 5 y old children.

Test		Pre-training	Post-training	Difference between post- and pre-training	*p* of Pre vs Post	*p* of Exp vs Con at post-training
***Posting coins-right hand (sec)***	Exp	22.79±4.43	22.00±5.73	-0.79±5.39	NS	NS
	Con	21.41±2.79	22.18±3.39	0.77±3.80	NS	
***Posting coins-left hand (sec)***	Exp	24.65±4.13	23.86±4.34	-0.79±3.37	NS	NS
	Con	24.72±4.20	23.70±4.15	-1.02±5.90	NS	
***Building Bricks (sec)***	Exp	27.76±8.52	28.77±6.09	1.10±8.45	NS	NS
	Con	25.66±5.50	28.33±9.35	2.67±9.01	NS	

*Abbreviations*: Exp = experimental group; Con = control group; NS = not significant; p = Bonferroni’s test. All data are expressed as mean±standard deviation. Raw data can be found in S1 Table

In all tasks, the values obtained at Pre-training were not statistically different between the Exp and the Con groups. At the end of the training period (Post-training) children of the Exp group performed significantly better in four out of six of the gross-motor-skill tasks: Putting a Medicine Ball, One Leg Balance on left foot, Balance on Beam and Balance on Platform (Table 1). The ability to maintain balance on the right leg was not modified by the training. For the Heel-to-Toe Walking test, there were no effects due to training; surprisingly, children of the Con group performed worse Post-training.

**Fig 3:**
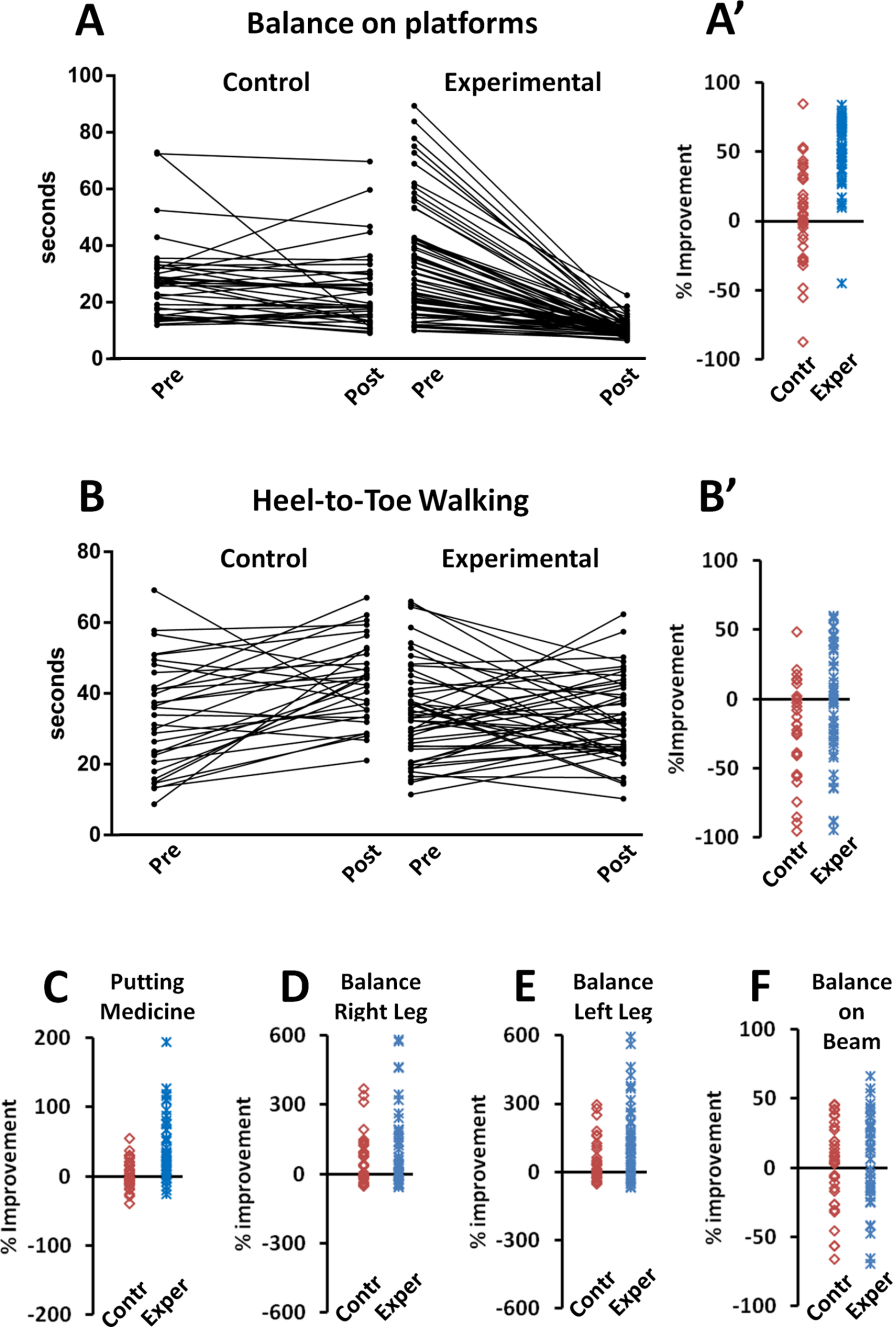
Individual outcomes of tasks used to test gross-motor skills. A: Balance on Platforms (time in seconds needed to walk along the six unstable platforms); B: Heel-to-Toe Walking (time in seconds needed to walk "Heel-to-Toe" on a 4.5 meter long paper tape on the floor without mistakes; C: Putting a Medicine Ball (distance in meter from a mark line); D: Balance on right Leg (time in seconds); E: Balance on left Leg (time in seconds); F: Balance on Beam (time in seconds required to walk along a 3.00 m beam). In A and B, individual pre- and post-training scores are connected by a line. In all panels, % improvements is given by the difference between scores expressed as % of the pre-training value; above the 0 value is improvement, below is worsening.

**Fig 4:**
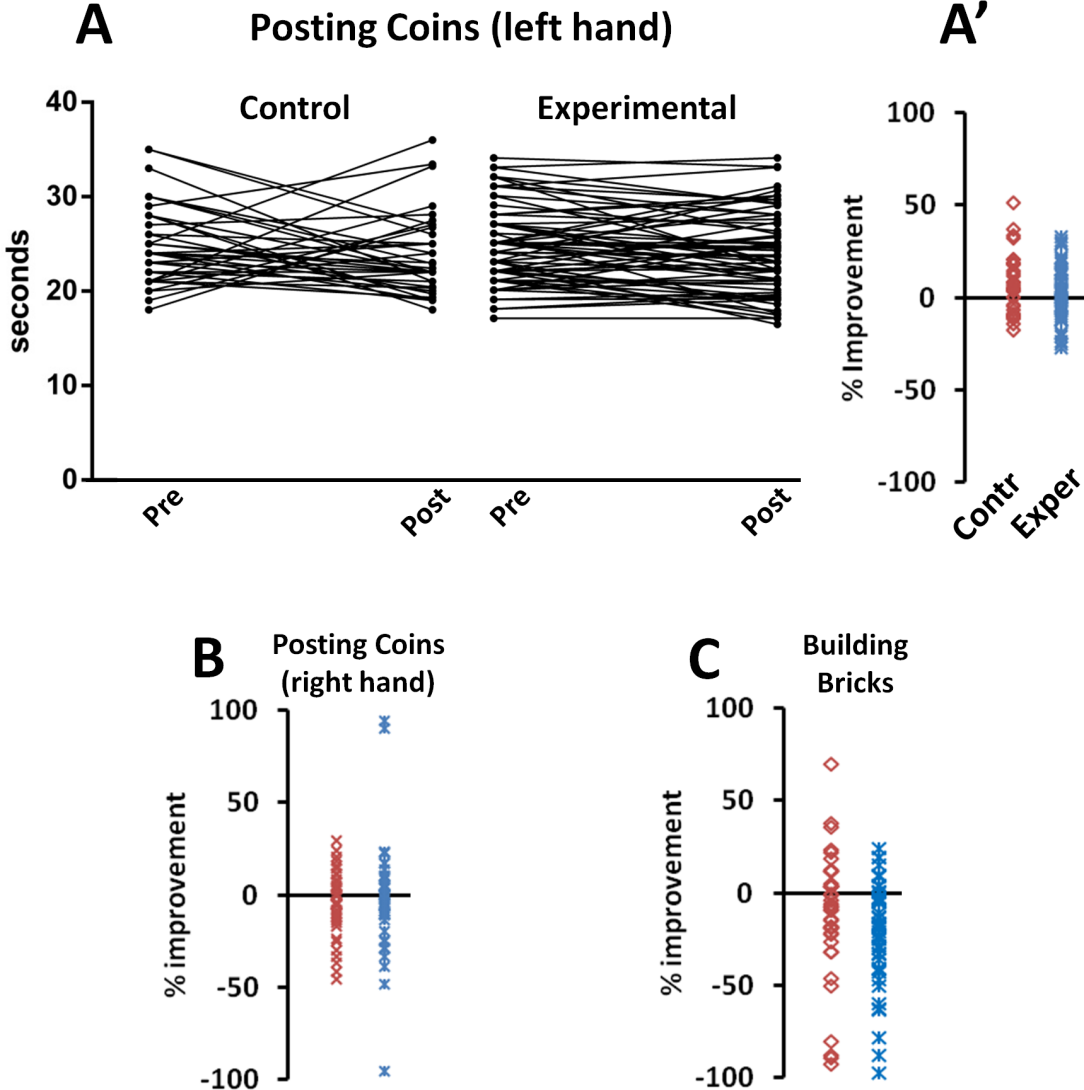
Individual outcomes used to test fine-motor skills. A: Posting coins with left hand (time in seconds to conclude the task; B: Posting coins with right hand (time in seconds to conclude the task); C: Building bricks (time in seconds to conclude the task). In A, individual pre- and post-training scores are connected by a line. In all panels, % improvements is given by the difference between scores expressed as % of the pre-training value; above the 0 value is improvement, below is worsening.

Individual subject performances usually varied between the two testing sessions for each task and the resulting mean values were due to improvement for some children and worsening for others (see examples in Fig 3A and 3B and Fig 4A). In 5 out of 6 tasks of gross motor skills assessment, more than half of the children of the experimental group performed better at the Post-training session (score > 10%) while this occurred for only 2 of the same tests for children of the Con group (data not shown). The extent of improvement (% variation respect to the Pre-training score) of each subject in the different tasks are shown in Fig 3A', 3B' and 3C–3F and Fig 4A', 4B and 4C. For the Exp group, the largest improvement occurred with the Balance on Platforms test (Fig 3A') and was minimal or absent for the Heel-to-Toe task (Fig 3B'). In the Balance on Platforms task, the improvement for the Exp group owned very high incidence (98.59% of children) and extent (average improvement of individual score was 59.1 for Exp vs. 5.9 for Con group). For the gross motor skill task that does not involve balance, i.e. Putting a Medicine Ball, both the fraction of children that performed better in the Post-training session (score improvement > 10%) and their individual improvement (% variation respect to the pre-training score) were larger for Exp vs. Con group (Table 1 and Fig 3C).

Mean data as well as individual variations measured for fine-motor skill tasks indicate that the 10 sessions of training did not significantly change the children capacity to perform the tests (Table 2 and Fig 4).

## Discussion

The present study explored the effects on motor skill competences induced in five year old children by playing structured and unstructured activities for one hour once a week for 10 times in a specific playground. The playground is located in northern Italy and the equipments present had been selected and aggregated on the basis of potential significance for basic motor skill development; only children under the age of 6 are allowed to enter the park [39]. The position and the physical characteristics of the equipments and the safety criteria concerning their use were constant throughout the study.

The experimental group improved significantly in four of the six tasks aimed at testing gross-motor skills: Balance on Beam, Balance on Platform, Putting a Medicine Ball and One leg balance (left foot). For a fifth task testing gross-motor skills, i.e. balance on right leg, the differences at the two time points were not significantly different; on the other hand the children of the experimental group showed a 43.3% improvement of their performance while children of the control group improved by 17.4% only. These results highlight the sensitivity of preschoolers to the effects of exposure to organized physical activities during school time and are in line with the study of Matvienko and Ahrabi-Fard [33] who found that a 4-week program consisting of daily morning walk and non-structured physically active play introduced significant improvements in motor skills and fitness levels compared to the control group.

Several evidences indicate that physical activity facilitates the development of motor competence in children [44–46]. Indeed practicing physical activities may involve locomotor skills (such as running, hopping and sliding), object control skills (such as throwing, catching and kicking) and stability skills (such as balancing, turning and twisting) [27, 41]. Reciprocally, preschool children that master different types of movements and have an extended motor repertoire, tend to be more physically active [47].

The issue of which type of physical activities should be included in the preschooler curriculum is an interesting issue. A general assumption is that specific training affects the development of some motor skill competences and not others [20, 48]; indeed, task-specific intervention with clumsy children reduced problems of movements related to the tasks that were actually trained [49]. As predicted by the Edelman's theory of "neural darwinism" that defines learning as a mechanism selecting neuronal networks on the basis of experience (training), it is expected that practicing with a specific tool has a direct impact on that task and not necessarily on other tasks related to the same competence [50–52]. Our data are in line with these theoretical premises. Indeed, the largest improvements induced by the training as compared to the control groups occurred with tests performed on equipments that were present in the park: balance beam and platforms. These equipments were available to children only during structured activities in the balance area (10 min for each visit). Thus, during the entire study, the children accumulated up to a total of 100 min of practice in the balance area, a time that appeared to be sufficient to improve their performance on those tasks. Interestingly, the training at the park had no effects on another balance-related task, the Heel-to-Toe Walking test, which was not practiced during the 10 visits at the park. Worth to note is that this last test does not involve elements of physical fitness such as muscle strength while it requires non-transferable and specific balance training that is not commonly performed during daily activities [53]. Thus, two closely related tasks related to balance, Walking on a balance beam and Heel-to-Toe walking along a line on the floor, had two different outcomes based on training suggesting that transfer between different exercises for the same motor competence is limited.

Another significant improvement associated to training in the park was found with the test Putting a Medicine Ball. This may be considered a gross motor task also related to aspects of physical fitness as it involves elements of muscle strength, endurance and coordination [42]. It is possible that the activities performed in the manuality and mobility areas of the playground may have influenced the outcome of this test. Indeed in these two areas the children of the experimental group had the opportunity to practice and train with tools such as monkey bars, hanging bar, climbing nets and ropes which are expected to have large impact on determinants of physical fitness (i.e. muscle strength). A similar "transfer effect" may have influenced the outcomes of the tests measuring the ability to stand on a single leg which is increased in the trained (even though statistically significant for one side only) and not in the control groups. In these tests both components of balance and leg strength are measured. Altogether the data suggest that transfer is high among some of the gross-motor activity competences when domains related to physical fitness and strength of specific muscle groups are involved (see also [54]).

As shown in the Results section, participation to the playground activities did not result in significant improvements of the performances in tasks measuring aspects of fine motor skills such as Building Bricks and Posting Coins (right and left hand). Since the exercise program in the playground focussed on gross motor skills, our result confirms that transfer of motor competences from gross- to fine-motor domains is limited (if not absent) and that improvement in the field of fine motor skills requires specific training [55, 56]. This aspect should be taken into consideration by educators of the Italian kindergarten where focus is on fine motor skills whereas attention to development of gross motor skills is limited [37].

## Conclusions

In conclusion this study revealed that the group who practiced gross motor activities in a specific playground in Treviso, Italy improved significantly in four out of six gross motor tasks compared to the control group. The organization of the activities associated to the peculiarities of the playground (organized in areas each dedicated to the exercise of a basic motor skill) and to the limited opportunities offered to Italian children to play actively in preschools probably contributed to generate significant improvements in the domain of gross motor skills even following a limited period of training.

During the study no training and opportunities were provided to specifically influence development of fine motor skills; lack of modifications was observed in the domain of fine motor skills (on specific tasks). Altogether our data provide important knowledge for educators when planning activities and organizing the environments dedicated to the promotion of healthy growth and motor development of children.

## Supporting Information

S1 Table S1 Table shows raw data used for the statistical analysis shown in Tables 1 and 2. (XLS)
